# The potential impact of PM_2.5_ on the covid-19 crisis in the Brazilian Amazon region

**DOI:** 10.11606/s1518-8787.2023057005134

**Published:** 2023-09-14

**Authors:** Karen dos Santos Gonçalves, Glauber G. Cirino, Marcelo Oliveira da Costa, Lucas de Oliveira do Couto, Giovane G. Tortelote, Sandra de Souza Hacon

**Affiliations:** I Barcelona Institute for Global Health Biomedical Data Science Team Barcelona Spain Barcelona Institute for Global Health. Biomedical Data Science Team. Barcelona, Spain; II Universidade Federal do Pará Instituto de Geociências Belém PA Brasil Universidade Federal do Pará. Instituto de Geociências. Belém, PA, Brasil; III World Wide Fund for Nature Brasília DF Brasil World Wide Fund for Nature. Brasília, DF, Brasil; IV Fundação Oswaldo Cruz Escola Nacional de Saúde Pública Sergio Arouca Rio de Janeiro RJ Brasil Fundação Oswaldo Cruz. Escola Nacional de Saúde Pública Sergio Arouca. Rio de Janeiro, RJ, Brasil; V Tulane University Department of Pediatrics New Orleans United States Tulane University. Department of Pediatrics. New Orleans, United States

**Keywords:** Covid-19, Brazilian Amazon, Wildfires, Fine Particulate Matter

## Abstract

**OBJECTIVE:**

This study aims to assess covid-19 morbidity, mortality, and severity from 2020 to 2021 in five Brazilian Amazon states with the highest records of wildfires.

**METHODS:**

A distributed lag non-linear model was applied to estimate the potential exposure risk association with particulate matter smaller than 2.5-µm in diameter (PM_2.5_). Daily mean temperature, relative humidity, percentual of community mobility, number of hospital beds, days of the week, and holidays were considered in the final models for controlling the confounding factors.

**RESULTS:**

The states of Para, Mato Grosso, and Amazonas have reported the highest values of overall cases, deaths, and severe cases of covid-19. The worrying growth in the percentual rates in 2020/2021 for the incidence, severity, and mortality were highlighted in Rondônia and Mato Grosso. The growth in 2020/2021 in the estimations of PM_2.5_ concentrations was higher in Mato Grosso, with an increase of 24.4%, followed by Rondônia (14.9%).

**CONCLUSION:**

This study establishes an association between wildfire-generated PM_2.5_ and increasing covid-19 incidence, mortality, and severity within the studied area. The findings showed that the risk of covid-19 morbidity and mortality is nearly two times higher among individuals exposed to high concentrations of PM_2.5_. The attributable fraction to PM_2.5_ in the studied area represents an important role in the risk associated with covid-19 in the Brazilian Amazon region.

## INTRODUCTION

Since the emergence of the first case of covid-19 in Wuhan, China in December 2019, the world has started to watch closely the growing number of cases in Asia and then its expansion worldwide.

Currently, covid -19 has ~670 million reported cases worldwide. Despite a high number of unnotified records, Brazil officially reports ~37 million cases (roughly 17% of its population), and a disturbingly high number of deaths, with roughly over 695 thousand lives lost since the beginning of the pandemic^1^. Brazil is still suffering from prolonged high levels of covid-19 mortality and inadequate pandemic responses, including little testing and disregard for scientific evidence^2^. Brazil is the largest country in South America, with a population of 214 million people^3^. Despite its privileged geographical location, which allowed the government to observe the pandemic unfold in other countries before reaching national territory, the country failed to implement an efficient plan to respond to the covid-19 pandemic, and has faced economic, social, and political crisis.

In 2021, from January to August, the country was at the peak of the pandemic crisis, with most of the states in the Brazilian Amazon in a precarious situation regarding medical and hospital care for covid-19 cases. For instance, in January 2021, the abrupt increase in the number of covid-19 hospital admissions in Manaus was unexpected and of huge concern worldwide^4^. Understanding the factors that might increase morbidity and mortality risk from covid-19 is essential for future prevention plans.

Several hypotheses and explanations for increasing covid-19 cases and deaths have been discussed, especially the factors that accelerate SARS-CoV-2 spread and the role of environmental factors, such as air pollutants^5^. Historically, the Brazilian Amazon region has been degraded by deforestation and wildfires^6^. Slash-and-burn farming is the most used method for clearing new areas for pasture, agriculture, or simply land speculation. However, from 2019 to mid-2021, the situation worsened, with nearly 11,000 km^2^ of forest loss in 2020, a 143% increase from 2012, when deforestation rates were recorded at the lowest values in the Brazilian Amazon^7^. This increase led to the emission of high concentrations of particulate matter (PM) along with other pollutants into the atmosphere, especially during the dry season. The concentration of PM_10_ (PM with diameters smaller than 10-µm) can reach up to 400μg/m^3 , 8^. In the Amazon biome, the PM_2.5_ (PM with diameters smaller than 2.5-µm) emissions from wildfires play a key role in air quality in the region. The inhalation and breathing of smoke from wildfires present adverse impacts on human health, including an increase in hospital admissions and premature mortality^9^, which leads to an overload of public healthcare services due to increasing cases of respiratory diseases, with worsening of the clinical condition, especially in the most vulnerable groups, such as pregnant women, children, older adults, firefighters, people with several comorbidities, and the Indigenous populations.

In recent years, several studies have shown that air pollution can be an important risk factor for adverse respiratory and cardiovascular health outcomes^10^. Studies regarding the epidemiological association of severe acute respiratory syndrome caused by covid-19 with PM_2.5_ exposure in the Brazilian Amazon region remain limited and unclear. Short- and long-term PM_2.5_ exposure has been extensively reported to contribute to systemic inflammation in healthy, non-smoker, and young populations^11^. This hypothesis indicates that PM_2.5_ exposure may increase vulnerability and lead to detrimental effects on the prognosis of patients affected by covid-19^12^. Therefore, PM_2.5_ exposures may contribute to a higher incidence of morbidity and mortality of covid-19 in the Amazon. A study assessing data from 355 Dutch municipalities to identify the relationship between concentrations of PM_2.5_, NO_2_, SO_2,_ and cases of covid-19 in hospital admissions have showed that an increase of 1 μ/m^3^ in the concentrations of PM_2.5_ is associated with 9.4 more cases of covid-19, 3.0 more hospital admissions, and 2.3 more hospitalizations^13^. Similar results were found in a study examining covid-19 hospitalizations in the USA, in which an increase of 1µg/m^3^ in PM_2.5_ was associated with an 8% increase in covid-19^14^. The current knowledge of the spread and infection mechanism of the SARS-CoV-2 can be summarized into three main hypotheses. First, aerosol particles and PM could prolong the duration of the airborne virus staying viable for hours, increasing the chance of transmission^15^. Second, PM may contribute to increasing the expression of angiotensin-converting enzyme 2 (ACE2) in the lungs and tissue susceptibility to SARS-CoV-2 infection^16 , 17^. Lastly, due to the fine particulate matter diameter, PM can directly transport the virus deep into alveolar and tracheobronchial regions^18^.

Considering the environmental health context in the region, an assessment was conducted to determine the potential exposure risk association between PM_2.5_ and covid-19. Notified cases, deaths, and hospital severe cases admissions were analysed in the five Amazon States with the highest records of fire hotspots, from February 25, 2020, to September 20, 2021.

## METHODS

### Study Design and Area: Brazilian Amazon States

For this study, an epidemiological retrospective analysis was conducted and PM_2.5_ emissions were estimated for five states in the Brazilian Amazon: Acre (AC), Rondônia (RO), Mato Grosso (MT), Para (PA), and Amazonas (AM). The study area presents heterogeneous characteristics, land use, climatic conditions, and socio-economic activities^19^. The studied areas encompass both the aerosol emissions due to forest degradation and urban and biogenic component^20^, as to the deforestation arc region at the south of the Amazon basin^21^. In broad terms, the most representative possible areas of PM_2.5_ emissions^22^ were selected both from local and regional fires, as well as urban emissions, plumes of pollution from neighboring regions, representative of the epidemiologic dataset assembled to analyze. In addition, this region is marked by high levels of poverty and inequality, being among the regions with the lowest Human Development Index (HDI-M) in the country, below 0.750.

### Data Sources

Covid-19 data were collected from public databases at the Johns Hopkins Coronavirus Resource Center website (https://coronavirus.jhu.edu), at the Coronavirus Brazil Panel of the Ministry of Health (https://covid.saude.gov.br/), and at the Flu Epidemiological Surveillance System (SIVEP-GRIPE) available by the covid-19 observatory network at the Oswaldo Cruz Foundation website (https://bigdata-covid19.icict.fiocruz.br/). All daily data were analysed for the study area from February 25, 2020, to September 20, 2021.

Information regarding the Brazilian population was obtained at the webpage of the Brazilian Institute of Geography and Statistics (IBGE) (https://ibge.gov.br/). Mean daily temperatures were collected from the National Meteorological Institute (INMET) (https://portal.inmet.gov.br) for the analysed period. Additional information on covid-19 community mobility reports monitored by GPS mobiles was collected from Google (https://www.google.com/covid19/mobility/) to understand the influence of restrictions during the pandemic. As an indicator of healthcare access, the number of hospital beds available was obtained from the Brazilian National Register of Health Establishments (CNES) from the Brazilian National Health System (SUS) available on Computer Department, Ministry of Health (DATASUS) (http://www2.datasus.gov.br/).

### Outcomes

The primary outcome was morbidity and mortality by covid-19 in and out-hospital. Total cases, deaths, and severity were analysed.

### Data Analysis: Remote Sensing of Aerosol Optical Depth and Spatialized Estimation of PM2.5

To achieve the PM_2.5_ predictions, the remote AOD estimates, from Satellites Terra (AQUA), were applied^23^. With the MOD043K (MYD043K) products, the newest algorithms and optical models, we are capable of estimating and monitoring the optical properties of atmospheric particles in the spatial resolution of 3 km, distinguishing various types of aerosols with good precision as terrestrial aerosol, marine, and horizontal dust from long distances^24^. The newest data collection available by Nasa (LAADS DAAC), Collection v. 6.1 (C6), which presents various enhancements concerning collection 5 (C5), was used^25^. The approach of PM_2.5_ estimation was based on the non-linear prediction model and the higher-resolution MODIS AOD products by modelling the AOD-PM_2.5_ relationship in a time-dependent manner reflecting seasonal fluctuations and influences of temperature and relative humidity with good fitting properties. This procedure was described by Paixão^26^and Gonçalves et al.^27^

### Data Analysis: Estimation of Potential Exposure Risk Association

A distributed lag non-linear model (DLNM) was applied to estimate the potential exposure risk association^28^. The statistical modelling simultaneously describes nonlinear and time-lagged dependencies between exposure to PM_2.5_ and the studied outcome, allowing extrapolations with the aid of regressions based on generalized linear models (GLM). This association is defined as an effect-lag-response type and demonstrates not only the exposure-response relationship but also the pattern of its structure over time and the exposure cumulative risk effects.

The relative risk (RR), attributable risk percent (ARP), both with a 95% confidence interval (95%CI), and population attributable risk percent (PAR%) were estimated in association with an increase of 1µg/m^3^ in the PM_2.5_ exposure concentrations. To control long- and short-term seasonality, the days of the week and holiday variables were applied fitting the model to the series centralization terms. To control meteorological confounding factors, the temperature and relative humidity were included. covid-19 is a new infectious disease and presents specific factors related to the spread of the virus. Information about community mobility and hospital beds to the model were added to control possible confounding factors related to physical distancing restrictions and healthcare access. The coefficient of determination (R^2^) was estimated to examine the differences between observed data and estimated data for the potential exposure risk to PM_2.5_.

The DLNM provides three analytical steps of regression analysis based on the Quasi-Poisson distribution, which aims to control the overdispersion of daily count data: 1) GLM using a natural spline, 2) GLM with Double threshold estimation, and 3) Simple GLM. Each regression was calculated assuming the cumulative risk effect of exposure to PM_2.5_ estimated concentration values for 7 days (lags = 7 days) and the time series divided by interquartile range (IQR) distribution (25^th^, 50^th^, and 75^th^ percentile) with three knots. The quasi-Poisson DLNM regression model was as follows:


 covid-19 ( morbidity, mortality, or severity )= intercept + dlnm ( pollution, lag =0−7 days )+ spline ( date )+ temperature + relative humidity + mobility + hospital beds + days of the week + holidays.

The estimation of attributable risk percent (ARP) considers the daily observed covid-19 data that can be attributed to potential PM_2.5_ exposure concentrations since it combines RR and prevalence of exposure. It is measured as ARP divided by the incidence risk in the exposed, according to the formula: AF=((Ie−Iu)/Ie) The population attributable risk percent (PAR%) helps determine which exposures are most important in each of the five states and it is calculated as the incidence of disease in the total state population, minus the incidence in the group of those unexposed to a specific risk factor (It−Iu) , thus giving the risk attributable to that risk factor in the population. It is measured as PAR=((It−Iu)/(It×100) , in which *It* is the incidence rate for the total population, and *Iu* is the incidence rate among the unexposed^29^.

The daily mean, median, maximum, and minimum values were calculated for the environmental variables (PM_2.5_ estimation concentration, temperature, and relative humidity). The daily data of covid-19 cases, deaths, and severe cases were applied in the models, and rates were calculated considering the constant of 10,000 inhabitants. The changes in the community mobility report were calculated in percentage (%) and the baseline was the median value for the corresponding day of the week, when the covid-19 pandemic started in Brazil (24–29 February 2020). The number of hospital beds was estimated in percentual changes (%). All the estimations and models were calculated in RStudio IDE^30^.

## RESULTS

### Descriptive Analysis

Overall, 1,308,711 cases, 37,263 deaths, and 104,875 severe cases of covid-19 were reported for the five states from February 2020 to September 2021. Table 1 shows the descriptive statistics specifically for the studies area. The table presents information regarding the number of municipalities; area extension (km^2^); population; mean, median, and maximum values; covid-19 incidence, mortality, and severity rates; and the percentual growth from 2020 to 2021. The covid-19 incidence, severity, and mortality were higher in Mato Grosso (1,641.7 notified cases; 110.4 severe cases; 42.4 deaths per 10,000 inhabitants), followed by Rondônia (1,512.3 cases; 93.0 severe cases; 37.2 deaths per 10,000 inhabitants), and Amazonas (1,099.1 cases; 97.3 severe cases; 35.4 deaths per 10,000 inhabitants).

**Table 1:** Descriptive statistics of five states in the Brazilian Amazon region from February 25, 2020, to September 20, 2021.

Variables	States of Brazilian Amazon region				

	Acre	Amazonas	Mato Grosso	Pará	Rondônia
Municipalities	22	62	141	144	52
Area (km^2^)	152,581	1,571,000	903,357	1,248,000	237,576
Population	790,101	3,874,000	3,224,000	8,074,000	1,749,000
Covid-19					
Notified cases	8,791	425,778	529,288	587,586	264,504
Deaths	1,816	13,709	13,674	16,567	6,512
Severe cases	4,664	37,706	35,579	56,774	16,264
Covid-19 rates (10k pop)					
Incidence	111.3	1,099.1	1,641.7	727.7	1,512.3
Mortality	22.9	35.4	42.4	20.5	37.2
Severity	59.1	97.3	110.4	70.3	93.0
Growth 2020–2021 (%)					
Incidence	28.4	59.4	102.5	30.1	158.4
Mortality	11.3	11.8	93.3	10.1	76.3
Severity	60.5	59.2	168.1	43.1	249.7
PM_2.5_ (µg/m^3^)					
Mean	8.2	8.1	9.1	7.5	9.3
Median	6.7	5.5	7.3	4.9	7.4
Max	46.8	74.1	68.6	50.0	49.6
Growth 2020–2021 (%)	10.4	7.7	24.4	10.4	14.9
Temperature (°C)					
Mean	27.5	27.5	27.7	26.9	27.6
Median	27.4	27.4	28.2	27.0	28.1
Max	32.2	32.1	36.7	30.2	36.6
Relative humidity (%)					
Mean	75.6	75.6	53.0	81.8	52.8
Median	76.9	76.9	54.4	82.3	54.2
Max	94.5	94.5	88.7	98.6	88.7
Community mobility growth 2020–2021 (%)	54	70	85	51	75
Hospital beds growth 2020–2021 (%)	8	6	3	9	9

Regarding the exposure variable, the highest maximum values of PM_2.5_ were estimated for the state of Amazonas (74.1µg/m^3^), followed by Mato Grosso (68.6µg/m^3^), Pará (50.0µg/m^3^), Rondônia (49.6µg/m^3^), and Acre (46.8µg/m^3^). The daily mean values were higher in Mato Grosso (9.1µg/m^3^) and remarkably similar among the other states, except for Para, which registered lower daily mean values (7.5µg/m^3^) from February 2020 to September 2021. The 2020/2021 growth in the PM_2.5_ concentration estimation was greater in Mato Grosso, with an increase of 24.4%, followed by Rondônia (14.9%). Considering the mean daily temperature, which was included for controlling confounding factors, the maximum values were highlighted in Mato Grosso (36.7°C), and Rondônia (36.6°C). The other values were remarkably similar in the other analyzed states. The state with the highest temperature daily mean values were Mato Grosso (28°C) and Rondônia (28°C), followed by Amazonas (27°C), Pará (27°C), and Acre (27°C).

The percentage change in community mobility from the beginning of the covid-19 pandemic to the present was higher in Mato Grosso, with an increase of 85% in mobility, followed by Rondônia (75%), Amazonas (70%), Acre (54%), and Pará (51%). It is important to highlight the lowest percentage growth from 2020 to 2021, which was registered in the state of Mato Grosso with 3%, followed by Amazonas (6%) representing a worrying and important piece of information about healthcare access in these states.

### Analysis of Potential Exposure Risk Association

Table 2 shows the overall relative risk (RR), attributable risk percent (ARP), population attributable risk percent (PAR%), and confidence intervals (95%CI) of potential exposure risk to an increase of 1µg/m^3^ in the estimated concentrations of PM_2.5_. The table also shows the p-values with statistical significance less than 0.0001. A significant statistical association between exposure to PM_2.5_ and covid-19 incidence, mortality, and severity with daily cumulative risk effects of 7 days was found in the five Brazilian states.

**Table 2:** Relative risk, attributable risk percent, population attributable risk percent, and confidence intervals (95%CI) of potential exposure risk to PM2,5 of five states in the Brazilian Amazon region.

Variables	States of Brazilian Amazon region				

	Acre	Amazonas	Mato Grosso	Pará	Rondônia
Covid-19 notified cases					
RR (95%CI)	1.5 (1.13–1.72)	1.8 (1.30–2.57)	1.6 (1.17–1.88)	1.8 (1.52–2.22)	1.4 (1.10–1.64)
ARP (95%CI)	0.87 (0.92–0.97)	0.79 (0.62–0.82)	0.83 (0.37 – 0.97)	0.88 (0.86–0.90)	0.87 (0.84–0.89)
PAR%	13	27	27	77	11
p-value^a^	0.000	0.000	0.000	0.000	0.000
Covid-19 deaths					
RR (95%CI)	1.8 (1.62–2.79)	1.5 (1.19–1.95)	1.8 (1.30–2.66)	1.9 (1.58–3.53)	1.1 (1.08–1.30)
ARP (95%CI)	0.56 (0.47–0.61)	0.57 (0.45–0.66)	0.86 (0.82–0.89)	0.81 (0.75–0.91)	0.61 (0.40–0.85)
PAR%	3	5	9	6	8
p-value	0.000	0.000	0.000	0.000	0.000
Covid-19 severe cases					
RR (95%CI)	1.4 (1.30–2.05)	1.6 (1.21–1.83)	1.6 (1.16–2.22)	1.6 (1.57–2.46)	1.2 (1.10–2.45)
ARP (95%CI)	0.53 (0.22–0.61)	0.68 (0.60–0.73)	0.65 (0.62–0.71)	0.77 (0.7 –0.79)	0.71 (0.44–0.77)
PAR%	15	21	25	12	22
p-value	0.000	0.000	0.000	0.000	0.000

RR: relative risk; ARP: attributable risk percent; PAR%: population attributable risk percent; 95%CI: 95% confidence interval.

The highest overall RR among covid-19 notified cases due to exposure to PM_2.5_ concentration was found for the states of Amazonas (RR = 1.8; 95%CI: 1.30–2.57) and Pará (RR = 1.8; 95%CI: 1.52–2.22), followed by Mato Grosso (RR = 1.6; 95%CI: 1.17–1.88), Acre (RR = 1.5; 95%CI: 1.13–1.72), and Rondônia (RR = 1.4; 95%CI: 1.10–1.64). The covid-19–PM_2.5_ attributable fraction was higher in all the states analysed (around 88%), except for Amazonas, which showed the lowest value (ARP = 79%). The population attributable risk percent (PAR%), used to assess the public health impact due to exposures to PM_2.5_ concentration in the populations analysed, was remarkably higher in Pará with 77%, followed by Mato Grosso, and Amazonas, both states with 27%.

Covid-19 mortality results showed the highest values of RR in the states of Pará (RR = 1.9; 95%CI: 1.58–3.53), Mato Grosso (RR = 1.8; 95%CI: 1.30–2.66), and Acre (RR = 1.8; 95%CI: 1.62–2.79). The ARP was higher in Mato Grosso (86%) and Pará (81%). The mortality PAR% showed a very similar pattern among the analyzed states. The results of covid-19 severe cases demonstrated the highest RR values in Mato Grosso, Pará, and Amazonas with relative risks of 1.6. The severity of ARP was relevant in the same states, but the PAR% showed the highest values in Mato Grosso, Rondônia, and Amazonas.

The percentage growth of community mobility indicated the adherence to covid-19 restrictions, such as lockdowns and social distancing, in each analyzed state ( Table 2 ). The increase in community mobility was higher among the states of Mato Grosso, Rondônia, and Amazonas. These results indicate fewer mobility restrictions considering the waves of infection from 2020/2021. The percentage of hospital beds was very similar in all states. However, it is important to highlight the lowest percentage values for Mato Grosso and Amazonas. These results could be linked to restrictions in the healthcare services, which probably influenced the increasing number of cases and deaths.

**Figure 1:**
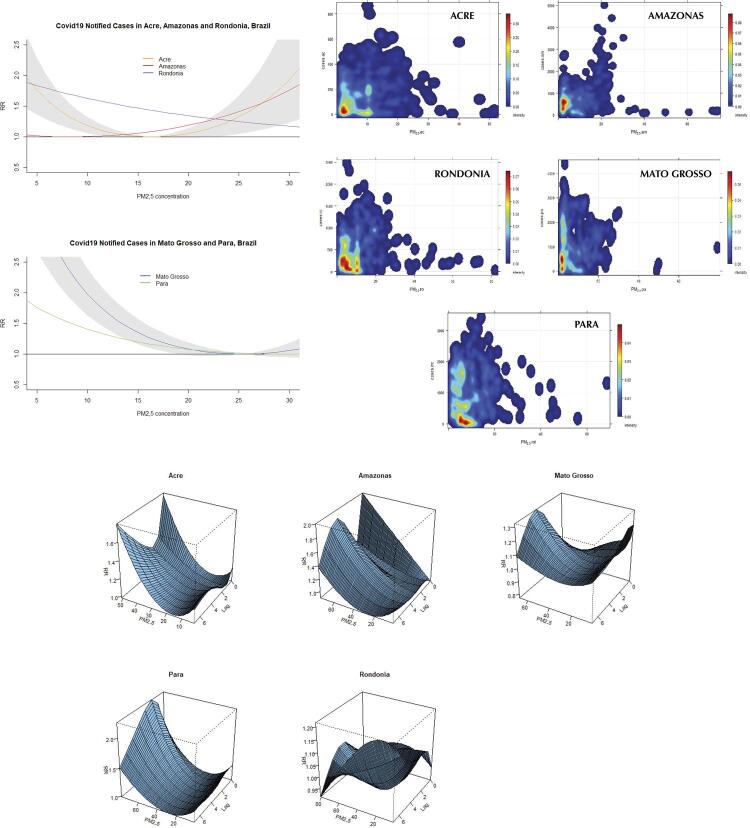
Overall risk effect of COVID-19 notified cases due to exposure to PM2.5 concentration in five states in the Brazilian Amazon region. Scatter plots show the relationship between the PM2.5 and the cases by state during the analyzed period. 3D plots show the dose-response effect of covid-19 cases by lags of 7 days.

^a^ p-value < 0.0001.

**Figure 2 f02:**
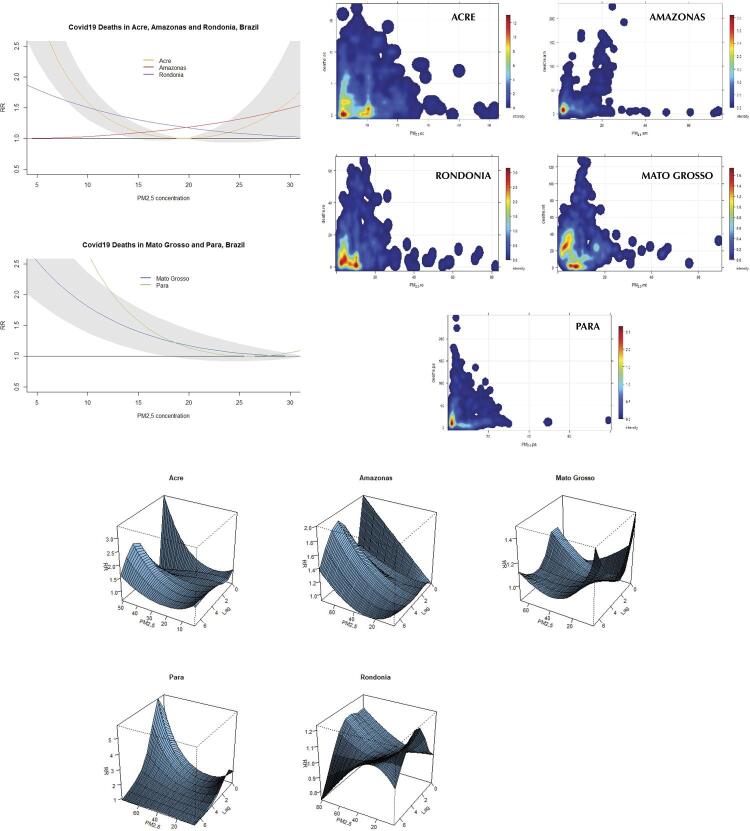
Overall risk effect of covid-19 deaths due to exposure to PM2.5 concentration in five states in the Brazilian Amazon region. Scatter plots show the relationship between PM2.5 and deaths by state during the analyzed period. 3D plots show the dose-response effect of covid-19 deaths by lags of 7 days.

**Figure 3 f03:**
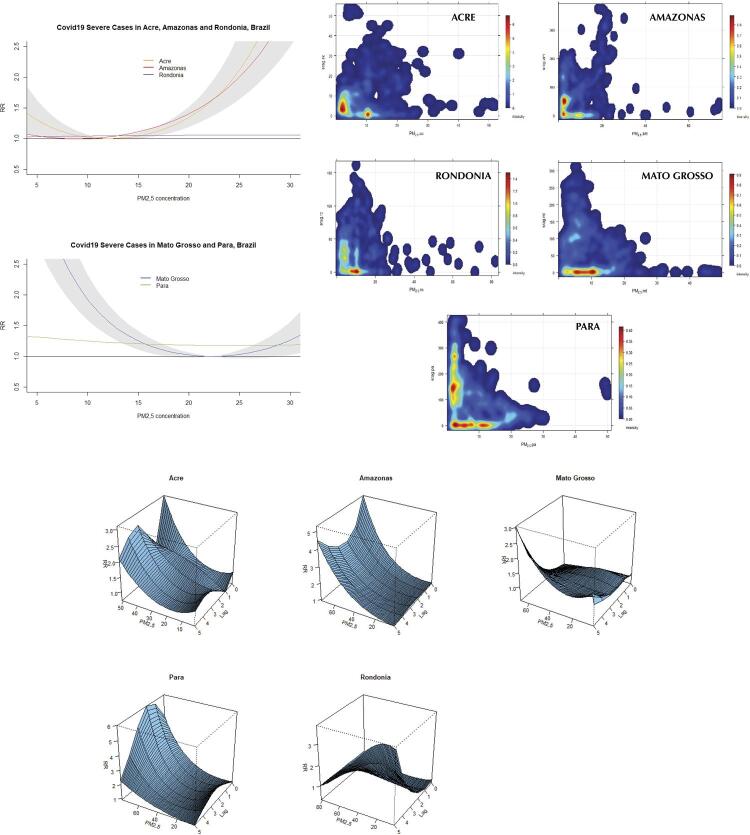
Overall severe cases of covid-19 in relation to exposure to PM2.5 concentration in five states in the Brazilian Amazon region. Scatter plots show the relationship between PM2.5 and deaths by state in the analyzed period. 3D plots show the dose-response effect of severe cases of covid-19 by lags of 7 days.

Figures 1–3 [Fig f02] [Fig f03] show the overall risk effect of covid-19 incidence, mortality, and severity due to exposure to PM_2.5_ concentration. The figures also show scatter plots with the relationship PM_2.5_–outcome, as well as 3D plots with the dose-response effect by lags of 7 days in the states and studied period. It is important to highlight that all RRs presented different outcomes of incidence, mortality, and severity, which were statistically significant differences, but showed ranges of maximum and minimum values with high variability, impacting the RR estimates. Interpreting the RR covid-19 19 incidence, the curve pattern of Amazonas and Acre showed an increased risk along with high PM_2.5_ concentration. Mato Grosso, Pará, and Rondônia, differently, showed a decreasing curve pattern due to the variability of a set of PM_2.5_ concentration values in the range of 20/30 µg/m^3^. The dose-response graphs showed the pattern of high values in the first 1–3 days lags, with a continuous increase until 7 days associated with PM_2.5_ concentration, except for the state of Rondônia, which showed a decrease. The results of covid-19 mortality and severity showed a similar curve pattern to the RR models described in the incidence. The exception was in severe cases, in which the states of Rondônia and Pará showed a static risk effect curve pattern, particularly concerning dispersion and variability of data.

## DISCUSSION

### Covid-19 and PM2.5 Exposure Risk Association

This study shows a relationship between air pollution regarding the particulate matter and the incidence, mortality, and severity of covid-19 in five Brazilian Amazon states. It also brings evidence about the possibility that the suspended particulate matter produced by wildfires has played a critical role in worsening the pandemic of covid-19 in the studied area.

In general, our findings showed that the risk of covid-19 infection, death, and severity is almost two times higher among individuals exposed to high concentrations of PM_2.5_. The attributable fraction to PM_2.5_ exposure represents an important factor in the risk associated with respiratory diseases in the Brazilian Amazon region. Therefore, PM_2.5_ exposure should be considered for the avoidance of critical situations and collapses in the public health of the region. Moreover, national and state controls over the deforestation rates should be strengthened towards the reduction of carbon emissions, consequently contributing to a climate balance and improved wellbeing.

The PM_2.5_ high concentrations emitted by slash-and-burn practices may have affected the SARS-Cov-2 spread in the Brazilian Amazon region, which explains the high relative risks found in this study. These results are according to forest fires distribution, which can contribute to more than 80% of the PM_2.5_ observed during the dry season in many regions of the Amazon basin, particularly in Bolivia and in the Brazilian states of Rondônia, Acre, and Mato Grosso^31^. We highlight that the maximum PM_2.5_ daily values found were higher than the acceptable value of PM_2.5_ per 24 hours (15µg/m^3^), recommended by the World Health Organization^32^.

The cumulative risk effects are following the literature when shown significantly positive RRs and 7 days of exposure to an outcome potential risk, following the time expected of covid-19 infection and the course of the disease of 7 to 14 days. In Spain, a longitudinal study assessed ambient air pollution, SARS-CoV-2 infection, antibody response, and covid-19. PM_2.5_ measured levels were associated with covid-19 with adjusted RR = 1.17 (95%CI: 1.03–1.32). Associations of PM_2.5_ with covid-19 were more pronounced for severe covid-19, with RR = 1.51 (95%CI: 1.06–2.16) for PM_2.5_^33^.

Considering the seasonality of wildfires practice in the Brazilian Amazon region, the findings were shown in a daily manner, aiming to find a relationship between high PM_2.5_ emissions concentrations during the dry season months and the increase of risk in covid-19 cases, deaths, and severity. The results suggest a hypothesis of a high association between the slash-and-burn practices and the increase in covid-19 incidence, mortality, and severity in the analyzed states. These states are among those with the highest records of fires registered by the Brazilian National Institute for Space Research (INPE)^34^. Our findings suggest that the highest values occur during the dry season months (from July 2020 to October 2020). Furthermore, it was possible to identify an increase in PM_2.5_ concentrations from January to April 2021, curiously coinciding with the period of the flexibilization in the social distancing restrictions in the states, also suggesting increased urban air pollution due to the increase of population mobility.

The interpretation of confounding factors due to the natural spread of a new infectious disease. such as covid-19. is important to demonstrate more robust findings. The model fitted by the percentage of community mobility and percentual hospital beds performed better with statistical adjustment. It is a good piece of evidence concerning the impact of air pollution on the incidence, mortality, and severity of covid-19, and is more consistent with the reality when considering the models’ important information, which could impact the correct interpretation of the results. These variables avoid the RR overestimation due to the data’s high variability.

Despite the literature presenting strong evidence of plausible biological mechanisms that could link exposure to air pollution with SARS-CoV-2^35^, our results indicate that air pollutants exacerbate symptoms and increase mortality rates for some respiratory diseases^36^. The RR differences found among Brazilian Amazon states may be better explained due to other factors, such as public health control measures adopted by each state and individual behaviors (e.g., social distancing, wearing masks).

In this context, our findings, conducted specifically in the Brazilian Amazon region, can provide valuable information as a planning tool for evaluating the effects of smoke exposure on human health. We believe that these findings can also allow the incorporation of alerts to public monitoring systems and, thus, promote real governance that integrates environmental and public health aspects.

### The Exposure Pattern in the Brazilian Amazon Region and Challenges of PM2.5 Estimation

The estimated PM_2.5_ results are explained in two aspects: (1) regional forest fires and (2) climatic phenomenon of natural variability. The first aspect strongly modulated the magnitude of PM_2.5_ values at the surface throughout the year, especially from July to October, which are months strongly influenced by deforestation and vegetation burning processes in the Amazon region; anomalous for the year 2020, especially in the *Pantanal* region of the state of Mato Grosso^37 , 38^. The PM_2.5_ regional distributions found are statistically compatible with surface measurements from previous experiments in Amazonia, not exceeding 5–10 µg m^-3^ during the rainy months, reaching values of 30–100 µg m^-3^ during the fires season. From July to October, our results show a 3-4 times growth in the biomass burning season, reaching mean values of ~30 µg m^-3^ in the studied areas, although values > 60 µg m^-3^ were frequently found in the western and southwestern portions of the states of Rondônia, Mato Grosso, and Pará, especially along its political borders and rivers present in the region^6 , 14 , 18 , 39^. In the case of the states of Amazonas and Acre, several local fires, located to the south and west of these states, respectively, may have also contributed to the increase in the PM_2.5_ mass concentration observed from July to October in those states. However, isolating the effect of local fires from regional transport on PM_2.5_ mass concentrations requires more sophisticated statistical analysis techniques and more information, outside the scope of this manuscript. This includes the transport of long-distance advected particles (such as particulates from the Sahara), which influence, to some extent, the concentration and mass of PM in the northern portions of the states of Pará and Amazonas. The second aspect, particularly important for the year 2020, refers to the monthly variation of PM_2.5_, which was also influenced by the La Niña phenomenon that traditionally induces drought regimes and strongly influences the distribution of PM_2.5_ in various regions of the Amazon^40^. We highlight that these two aspects eventually coexist during a given year, causing a big boost in the number of fires and, consequently, in PM_2.5_ emission in the Legal Amazon, as in the year 2020.

### Limitations of the Study

This study presents some methodological limitations that deserve to be highlighted. The estimated PM_2.5_ concentration values are from Nasa satellite images and uncertainties in resolution and optical depth capture of aerosols are possible. In this study, it was possible to validate the estimated series of PM_2.5_ with the series measured by an air quality monitoring station in the state of Acre. However the same validation for the other analyzed states was impossible.

The results may be understated since the health impact of fires has extended far beyond these cases. Many people in the Amazon were not even able to reach a healthcare service due to living in remote areas and requiring transportation by the river, or due to saturated healthcare services that could not provide care for covid-19. The impact may not be representable by hospitalization data since many people in Amazon have limited access to healthcare. Available data also excludes private healthcare services, although a quarter of Brazilians have private healthcare plans and tend to seek care in these establishments. Moreover, the data did not include the great number of people whose respiratory problems did not require hospitalization, despite being serious.

Other factors were not considered, which limit evidence from our study, such as the social determinants of health, access to healthcare services, quality of treatment used for covid-19, the patients’ social conditions, comorbidity prevalence, environmental factors, public policies, among others.

It is not possible to guarantee that the analyzed series of PM_2.5_ concentrations represented exclusively the smoke from the forest fires. Models for this estimation are more complex, and the values presented here must consider different sources of exposure, including urban exposure by mobile and fixed sources. However, by comparing PM_2.5_ values in drought periods and other periods without fires, it is evident that the high concentrations of PM_2.5_ are due to forest fires.

The health data comes from tracking “Big data” on different available bases, which may show a discrepancy between them to a greater or lesser extent. Since this is an ongoing pandemic that depends on screening and confirmation of positive cases, underreporting is possible, especially in the northern region of Brazil, where our study was conducted.

## CONCLUSIONS

The current epidemiological situation of the covid-19 evolution in Brazil is of global concern, notably due to the spread of the Omicron plus variant and the growing number of fire outbreaks registered for 2021 in Brazil, which could increase the severity of covid-19 in the Amazon region. The Brazilian healthcare system have showed a limited capacity to treat severe cases of covid-19 and respiratory diseases, including severe acute respiratory syndromes. The Brazilian Amazon region requires urgent actions toward the healthcare system, enabling it to effectively respond to crises like the covid-19 pandemic. Social distancing should also be implemented as a response to disease peaks, thus allowing for better control and coping with the covid-19 expansion in the country.

The relative risks observed in this study are high and point to a tremendous challenge in controlling the disease and the pressure on the entire healthcare system of the region. In addition to being a new infectious disease in progress and without an immunity group necessary to prevent its rapid progression, the situation becomes even more serious as the population is exposed to high concentrations of PM_2.5_, increasing the risk of illness and doubling the risk of mortality.

The covid-19 pandemic situation is even more challenging when considering the current and complex social, political, and economical scenario in Brazil, which leads to increasing forest loss in the Brazilian Amazon region and, consequently, growing emissions.

Our findings showed that the risk of covid-19 incidence, mortality, and severity is almost two times higher among individuals exposed to high concentrations of PM_2.5_. The attributable fraction to PM_2.5_ represents an important role in the risk associated with the Brazilian Amazon region. Therefore, actions and governance from decision-makers should be planned to avoid critical situations of chaos in the healthcare services of the region. The PM_2.5_ high concentrations due to deforestation may have affected the SARS-Cov-2 spread in the Brazilian Amazon region, which explains the high relative risks found in this study.
